# Interferon Regulatory Factor Family Genes: At the Crossroads between Immunity and Head and Neck Squamous Carcinoma

**DOI:** 10.1155/2022/2561673

**Published:** 2022-05-26

**Authors:** Shaokun Liu, Zhenlin Wang

**Affiliations:** Department of Otorhinolaryngology Head and Neck Surgery, Xuanwu Hospital Capital Medical University, No. 45 Changchun Street, Xicheng District, Beijing 100053, China

## Abstract

**Objective:**

This study is aimed at investigating the regulating mechanisms of the interferon regulatory factor (IRF) family genes in head and neck squamous cell carcinoma.

**Methods:**

Based on the HNSC data in the ‘The Cancer Genome Atlas (TCGA)' database, the expression pattern of IRF family genes was investigated. The association of IRFs family genes and survival outcomes were analyzed by Kaplan–Meier plotter web portal. The relation of IRF genes and tumor stages was evaluated by using stage plots and based on GEPIA portal. 50 genes interacting with IRFs were identified using the NetworkAnalyst's protein-protein interaction (PPI) network construction tool. The top 200 correlated genes with similar expression patterns in HNSC were obtained by the similar gene detection module of GEPIA. Furthermore, functional enrichment analysis was performed to determine the biological functions enriched by the interacting and correlated genes. The potential implication of IRFs in tumor immunity was investigated in terms of tumor-infiltrating immune cells, a pair of immune checkpoint genes (CD274 and PDCD1), and ESTIMATE-Stromal-Immune score.

**Results:**

The unpaired sample analysis shows that all of the IRF family genes were highly expressed in HNSC tumor samples compared to control samples. The survival analysis results showed that the overexpression of IRF1, IRF4, IRF5, IRF6, IRF8, and IRF9 was associated with better overall survival in HNSC, while the other IRFs genes (IRF2, IRF3. and IRF7) did not show prognostic values for overall survival outcome of HNSC. Four genes (STAT1, STAT2, FOXP3, and SPI1) were overlapping among 50 interacted genes in the PPI network and top 200 correlated genes identified by GEPIA. The 50 interacting genes in the PPI network and top 200 correlated genes were integrated into 246 genes. These 246 genes were found to be overrepresented in multiple KEGG pathways, e.g., Th17 cell differentiation, T cell receptor signaling pathway, cytokine-cytokine receptor interaction, natural killer (NK) cell-mediated cytotoxicity, FOXO signaling, PI3K-Akt signaling, and ErbB signaling. Most correlations between IRF gene members and TIICs were positive. The strongest positive correlation was identified between IRF8 and T cells (*r* = 0.849, *p* < 0.001). The majority of correlation between IRF family genes and ESTIMATE-Stromal-Immune score was found to be positive. The highest positive correlation was found to be between IRF8 and Immune score (*r* = 0.874, *p* = 1.09*E* − 158). Most correlations between IRFs and two immunoinhibitor genes (CD274 and PDCD1) were positive. IRF1 and PDCD1 were found to show the highest positive correlation (*r* = 0.764, *p* < 2.2*e* − 16).

**Conclusions:**

The current analysis showed IRFs were differentially expressed in HNSC, indicated significant prognostic values, were involved in tumor immunity-related signaling pathways, and significantly regulated tumor-infiltrating immune cells. IRF family genes could be potential therapeutic biomarkers in targeting tumor immunity of head and neck cancer.

## 1. Introduction

Interferon regulatory factors (IRFs) are well-known to be transcription factors involved in the regulation of interferon genes [1]. The interferon regulatory factor (IRF) family in mammals consists of nine members: IRF1, IRF2, IRF3, IRF4, IRF5, IRF6, IRF7, IRF8, and IRF9 [2]. Interferon regulatory factors (IRFs) are required for the activation of innate immune responses in response to pathogens and the subsequent induction of adaptive immunological responses [3]. It is believed that dysregulation of IRF signaling has a key role in the pathogenesis of autoimmune diseases, due to its inappropriate regulating role in immune cell activation and differentiation [3]. Although it was originally believed that IRFs function largely in the immune system by contributing to innate immune response, recent discoveries based on increasing evidence have revealed that IRFs play key roles in the governing oncogenesis [4–6]. Several outstanding and detailed reviews [4–6] have examined the role of IRFs in immunological disorders and malignancies in detail. They concluded that IRFs are placed at the nexus of immunity and cancer, connecting the processes governing both.

Increasing evidence has shown the role of IRF family genes in activating antitumor immunity and inhibiting immunosuppression. IRF1 was found to regulate C-X-C motif chemokine 10 (CXCL10)/chemokine receptor 3 (CXCR3) signaling axis, thereby further activating antitumor immunity in hepatocellular carcinoma (HCC). IRF4 was found to negatively regulate the development of myeloid-derived suppressor cells (MDSC) and its immunosuppressive function in tumors [7]. In addition, IRF family genes were found to be implicated in the antitumor immunity by controlling the differentiation and maturation process of tumor-associated immune cells including dendritic cells, NK cells, B lymphocytes, and T lymphocytes. For instance, IRF8 was shown to suppress tumor progression by inducing the maturation and differentiation of antigen-presenting cells (APCs) (e.g., MФ, DCs, and B lymphocytes) [8]. IRF4 was found to regulate the apoptosis of B lymphocytes by targeting the Fas apoptosis inhibition molecule [9]. Based on all this evidence, IRF family genes should be regarded as key mediators in regulating tumor immunity and thus might provide potential strategies in cancer immunotherapy.

Squamous cell carcinoma of the head and neck (HNSC) is the sixth most prevalent type of cancer worldwide, with a 5-year survival rate of less than 50% [10]. Previous studies demonstrated that IRF family genes were aberrantly expressed and also identified as diagnostic and prognostic markers in various hematological malignancies such as colorectal cancer [11], breast cancer [12], pancreatic cancer [13], and gastric cancer [14]. However, the effect of IRF family genes remains largely unclear in HNSC. The roles of IRFs in HNSC have not yet been investigated by using a systematic approach such as bioinformatics analysis. To the best of the authors' knowledge, this is the first study to examine the prognostic value and regulatory role of IRF4 especially focusing on immunity involvement.

The link between IRF family gene mRNA expression and clinical features of HNSC patients was studied using genomic and clinical data from The Cancer Genome Atlas (TCGA) database. Furthermore, the significant value of IRF family genes in HNSC prognosis was evaluated. In addition, functional enrichment analysis was used to determine IRF4's putative biological functions in HNSC. More importantly, the relationship between IRF4 and immunity was evaluated from several significant aspects including TIICs (tumor-infiltrating immune cells), a pair of classic immune checkpoint genes comprising of a receptor and its ligand (PD1 and PDL1), as well as the tumor microenvironment. The current research on IRFs may give a theoretical foundation for their mechanistic roles in tumor formation and immunology, as well as guidelines for medication therapy selection.

## 2. Methods

### 2.1. Study Design of the Present Research

**Figure 1:**
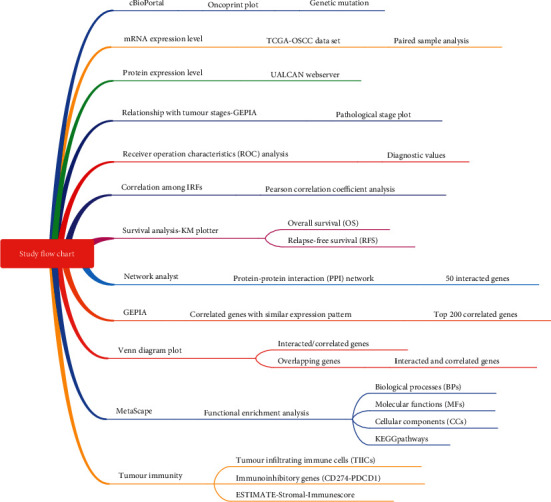
The schematic illustration of this study.

Figure 1 used a flowchart to show the study design of the current research. Firstly, the genetic mutation, mRNA expression, protein expression, relation to tumor stages, and prognostic values of IRF family genes in HNSC were investigated. Afterwards, the interacted genes and correlated genes of the IRF family genes were, respectively, identified. After integrating the interacted and correlated genes, this group of genes were used for the functional enrichment analysis. More importantly, tumor immunity involvement of IRFs in HNSC was analyzed by investigating three aspects including tumor-infiltrating immune cells, two classic immunoinhibitory genes, and ESTIMATE-Stromal-Immune score.

### 2.2. cBioPortal Analysis

The cBioPortal (https://www.cbioportal.org/) for Cancer Genomics is a free and open-source software platform that allows users to easily visualize and analyze genomic datasets on cancer at a large scale. The present study included results from 3 published HSNC studies: [1] TCGA, Nature 2015: 279 tumor samples; [2] TCGA, Firehose Legacy: 530 tumor samples; and [3] TCGA, PanCancer Atlas: 523 tumor samples. The cBioPortal analysis tool (version v1.11.3) was used to summarize the probable genetic changes of the nine IRF family genes in HNSC.

### 2.3. Unpaired and Paired Sample Analyses

The HNSC (head and neck squamous cell carcinoma) project retrieved RNAseq data in level 3 HTSeq-FPKM format from the TCGA (URL: https://portal.gdc.cancer.gov/) database. The FPKM (fragments per kilobase per million) format of the RNAseq data was translated to TPM (transcripts per million reads) and log2 transformed. The current study contained 546 samples, 502 of which were HNSC tumor samples and 44 of which were healthy control samples. The mRNA expression of nine IRF family genes was examined and shown in TCGA-HNSC data using the R package ggplot2. We conducted both unpaired and paired sample analyses.

### 2.4. Receiver Operating Characteristic (ROC) Curve Analysis

Receiver operating characteristic (ROC) analysis is a widely used technique in clinical epidemiology for evaluating the effectiveness of diagnostic tests for binary classification based on the distribution of tumor markers. The area under curve (AUC) value is a frequently used as an indication of test accuracy. A ROC plot running through the upper left corner indicates perfect discrimination, showing 100 percent sensitivity and 100 percent specificity. The closer the receiver operating characteristic plot is to the upper left corner, the greater the AUC, and the test is generally more accurate. The quantitative value for the AUC to segregate the quality of a classifier is as follows: AUC: 0.9-1 denoting excellent; 0.8-0.9 denoting very good; 0.7-0.8 denoting good; 0.6-0.7 denoting average; and 0.5-0.6 denoting poor. The diagnostic value of each IRF mRNA expression in HNSC was evaluated by plotting the ROC curve.

### 2.5. Correlation Analysis of IRF Family Genes

To determine the association between each pair of IRF family genes, a Pearson correlation coefficient (PCC) study was done. The expression level of each IRF gene was determined in TCGA-HNSC tumor samples. Between each pair, the *r* and *p* values were determined. Following that, a heatmap was created using the ggplot2 package (version 3.3.3) in the R software (version 3.6.3).

### 2.6. Protein Expression of IRF Family Genes in Primary HSNC Tumor Samples Compared with Healthy Control Samples

As an integrated data-mining platform, UALCAN (URL: http://ualcan.path.uab.edu/index.html) facilitates a comprehensive analysis of the cancer transcriptome. This web-based platform's functionalities enable users to analyze relative expression between tumor and normal samples for a query gene or multiple genes. The International Cancer Proteogenome Consortium (ICPC) datasets and the Clinical Proteomic Tumor Analysis Consortium (CPTAC) data are used by UALCAN to analyze protein expression. In addition, protein expressions are available for colorectal cancer, breast cancer, ovarian cancer, clear cell renal cell carcinoma, uterine corpus endometrial carcinoma, gastric cancer, glioblastoma, pediatric brain tumors, head and neck squamous cell carcinoma, lung adenocarcinoma, lung squamous cell carcinoma, liver cancer, pancreatic cancer, and prostate cancer. With UALCAN, users can export gene and protein expression and survival analysis results into graphical images that are publication-ready in png, jpeg, and PDF formats. The protein expression of IRF family genes in primary HNSC tumor samples compared with healthy control samples were analyzed in this webserver, and the statistical significance was calculated.

### 2.7. Kaplan–Meier Plotter (KM Plotter) Database for Survival Analysis

The KM plotter (http://kmplot.com/) was used to determine the effect of 30 k genes on survival in 21 different forms of cancer by using gene arrays, RNA sequencing, or next-generation sequencing. The datasets were constructed using data from GEO, EGA, and TCGA. The KM plotter database contains information on the survival of 500 patients with HNSC. In the KM plotter online database, the prognostic relevance of IRF family members were assessed. The Kaplan–Meier curves were used to determine the link between target gene mRNA expression levels and relapse survival rate (RFS) and overall survival (OS) rates in the HNSC tumor group. The Kaplan–Meier survival graphs were used to illustrate the results. Using an online tool, the hazard ratio (HR) and 95% confidence interval (CI) were calculated automatically. The mean standard deviation is used to express the values for each group. When the Log-rank test was applied, a *p* value of 0.05 was considered statistically significant.

### 2.8. The Relationship between IRF Family Genes and Tumor Stages

The expression DIY module of the web platform Gene Expression Profiling Interactive Analysis (GEPIA) (http://gepia.cancer-pku.cn) was used to plot gene expression by tumor stage, based on the TCGA clinical annotation. The *F* test was used to determine the expression of IRFs in various stages of HSNC tumors. The pathological stage map was created by using GEPIA platform to compare the expression of a certain IRF family member in various stages of HSNC tissue. *p* < 0.05 was judged as statistically significant.

### 2.9. NetworkAnalyst Webtool Analysis

NetworkAnalyst (URL: https://www.networkanalyst.ca/) is a visual analytics platform for comprehensive gene expression profiling and meta-analysis. Firstly, the organism was specified to be H. sapiens (human), and Entrez ID of all IRF family genes were uploaded: 3659, 3660, 3661, 3662, 3663, 3664, 3665, 3394, and 10379. Secondly, generic protein-protein interactions (PPI) was selected to be constructed based on the STRING interactome database. The confidence score cutoff was set as 900, and all the interactions required experimental evidence. Afterwards, a network comprising of 59 nodes, 87 edges, and 9 seeds was built and viewed.

### 2.10. Similar Gene Detection Analysis

A similar gene detection module in the GEPIA webtool was used for identifying genes that have a similar expression pattern with a gene signature (IRF1-9) in the HNSC cancer type. The top 200 correlated genes were identified according to the descending order of the Pearson correlation coefficient (PCC) value. According to the interpretation of the Pearson correlation coefficients defined in the medicine area, |*r*| value: 0: none; >0-0.3: poor; 0.3-0.6: fair; 0.6-0.8: moderate; >0.8: very strong; and 1: perfect.t

### 2.11. Venn Diagram Analysis

In the previous steps, the 50 genes interacting with IRFs were identified by building a PPI network by using NetworkAnalyst, and the top 200 genes highly correlated with the IRF gene signature were identified by using the GEPIA webtool. A Venn diagram webtool (URL: http://bioinformatics.psb.ugent.be/webtools/Venn/) was used to identify the gene list which were overlapped between these two groups of genes. This tool is able to calculate the intersections of list of gene elements. The correlations between the overlapping genes and IRFs were identified and shown in a heatmap.

### 2.12. Metascape Analysis

Metascape database (URL: https://metascape.org) is able to combine functional enrichment and gene annotation into a single integrated gateway, leveraging over 40 separate knowledge bases. The IRFs-interacted genes and IRFs-correlated genes were integrated and the union genes of these two groups were obtained. This gene list was uploaded in the Metascape webtool, and custom enrichment analysis was performed. Four functional sets (GO biological processes (3683), GO molecular functions (577), GO cellular components (391), and KEGG pathways (212)) were, respectively, selected.

### 2.13. Tumor-Infiltrating Immune Cell (TIIC) Analysis

Pearson correlation coefficient analysis was used to determine the relationship between IRF family genes and tumor-infiltrating immune cells in HNSC tumor samples. The statistical method used the ssGSEA algorithm from the GSVA package (version 1.34.0). The lollipop plot was used to illustrate the link between the expression of IRFs and 24 different types of TIICs in HNSC samples.

### 2.14. ESTIMATE-Stromal-Immune Score

An analysis was carried out to analyze the correlation between IRF family genes and tumor immune microenvironment (TIME). Researchers recently developed an algorithm for detecting infiltrating stroma and immune cells based on gene expression signatures called ESTIMATE (Estimation of Stromal and Immune Cells in Malignant Tumor Tissues Using Expression Data). Based on the ESTIMATE algorithm, the correlation between IRF family genes and ESTIMATE-Stromal-Immune score were calculated in HNSC.

### 2.15. TISIB Webtool Analysis

TISIDB (http://cis.hku.hk/TISIDB/) is a web-based comprehensive database for tumor-immune system interactions that incorporates a variety of data types. This web portal was used to investigate the relations between two typical immunoinhibitor genes (PDCD1 and CD274) and expression, copy number, methylation, or mutation of each specific IRF gene. The reason of selecting these two genes is based on the fact that programmed cell death-1 (PDCD1) and its ligand programmed cell death 1 ligand 1 (CD274/PDL1) have been established to implicated in the T cell-mediated suppression of antitumor immunity.

## 3. Results

### 3.1. Genetic Alterations of IRF Family Genes in HNSC

**Figure 2:**
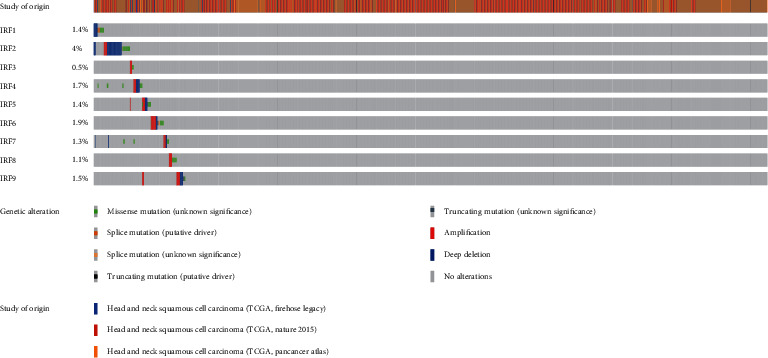
Oncoprint plot shows the genetic alterations of 9 IRF family genes in HSNC.

The OncoPrint plot was generated to analyze genetic alterations (Figure 2). The distribution of each IRF family gene's genomic alterations in the TCGA HNSCC dataset is shown as follows: IRF1 (1.4%), IRF2 (4%), IRF3 (0.5%), IRF4 (1.7%), IRF5 (1.4%), IRF6 (1.9%), IRF7 (1.3%), IRF8 (1.1%), and IRF9 (1.5%).

### 3.2. The Dysregulation of IRF Family Genes in HNSC

**Figure 3:**
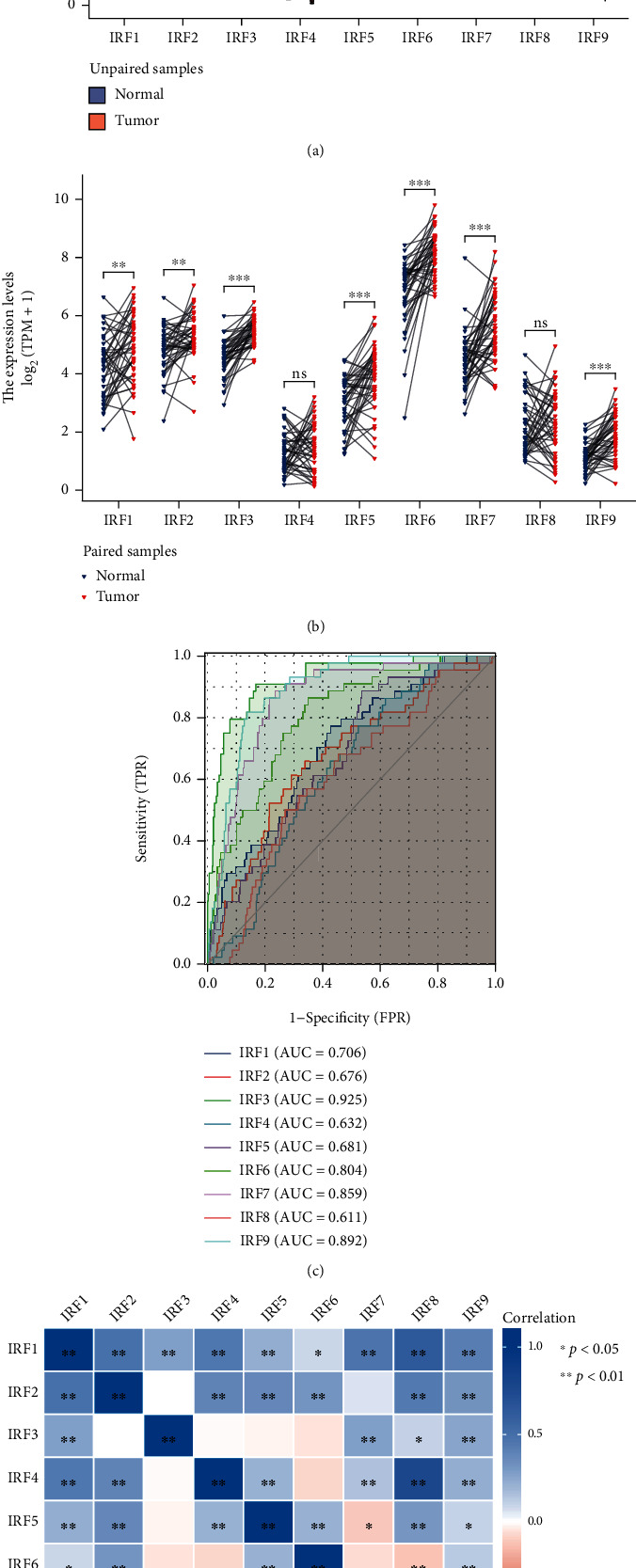
(a) The expression pattern of IRF family genes in HNSC by performing unpaired sample analysis. (b) The expression pattern of IRF family genes in HNSC by performing paired sample analysis. (c) ROC curve used for evaluating the diagnostic values of IRF family genes in predicting the tumor status of HNSC. (d) The heatmap showing the correlation between IRF family genes in HNSC.

The unpaired sample analysis was based on 546 samples containing 502 HNSC tumor samples and 44 healthy control samples. The results showed that the samples did not pass the normality test (*p* < 0.05); thus, the Mann–Whitney *U* test (also named as Wilcoxon rank sum test) was adopted for this investigation.  Figure 3(a) shows that the mean expression levels of all IRF genes were significantly greater in HNSC tumor samples than in healthy control samples (*p* < 0.05).

The paired sample analysis was based on the 43 HNSC tumor samples and their adjacent 43 healthy control samples. The results revealed that the differences between the tumor and control groups did not meet the criteria of the normality test (*p* < 0.05); thus, the Wilcoxon signed rank test was used for the current study.  Figure 3(b) shows that the expression level values of all IRF genes in the HSNC tumor sample group were greater than that in the healthy control sample group. There was no statistically significant difference in IRF4 and IRF8; however, a statistically significant difference was observed for the other genes.

### 3.3. Diagnostic Value of IRF mRNA Expression in HNSC

The results (Figure 3(c)) showed that the AUC value of each IRF gene was, respectively, as follows: IRF1: AUC = 0.706 (good); IRF2: AUC = 0.676 (average); IRF3: AUC = 0.925 (excellent); IRF4: AUC = 0.632 (average); IRF5: AUC = 0.681 (average); IRF6: AUC = 0.804 (very good); IRF7: AUC = 0.859 (very good); IRF8: AUC = 0.611 (average); and IRF9: AUC = 0.892 (very good).

### 3.4. Correlation between the IRF Family Genes in HNSC

The correlation analysis results in Figure 3(d) and Table 1 indicated that there was a strong correlation among the IRF family genes. There was a significant positive correlation among most IRF genes. The negative correlation was observed in the following pairs: IRF5 and IRF7 and IRF6 and IRF8.

### 3.5. Dysregulated Protein Expression of IRF Family Genes in HSNC

**Figure 4:**
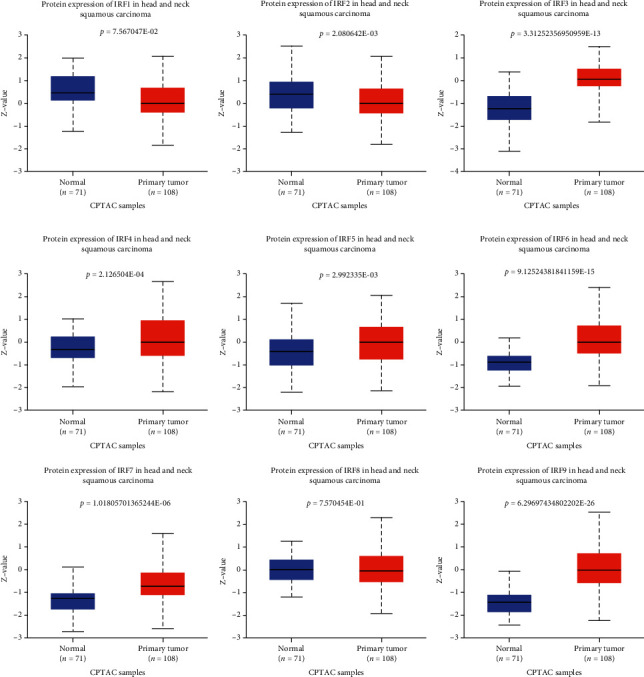
The protein expression of all IRF family genes in head and neck squamous carcinoma. *Z*-values represent standard deviations from the median across samples for the given cancer type. Log2 Spectral count ratio values from CPTAC were first normalized within each sample profile and then normalized across samples.

Figure 4 shows that the protein level of IRF1, IRF2, and IRF8 were significantly downregulated in HNSC compared with healthy control samples. Except for these three genes, the protein level of the other IRF family genes (IRF3, IRF4, IRF5, IRF6, IRF7, and IRF9) were significantly upregulated in HNSC compared with healthy control samples.

### 3.6. The Prognostic Values of IRF Family Genes in HNSC

**Figure 5:**
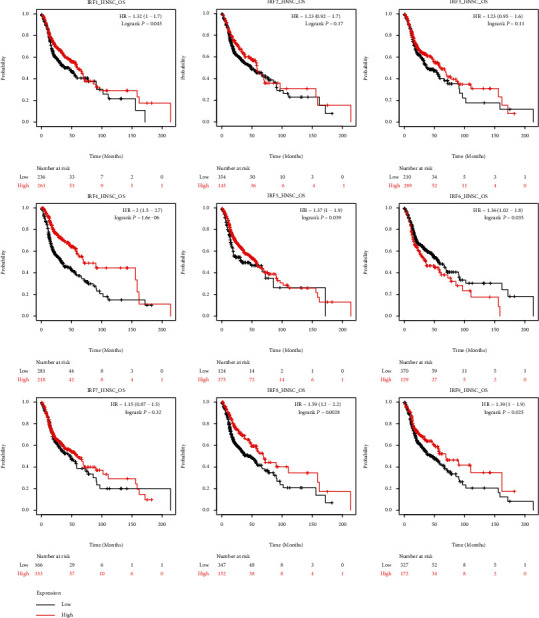
Kaplan–Meier survival curves for visualizing the association of overall survival (OS) and expression of IRF family genes in TCGA_HNSC data.

**Figure 6:**
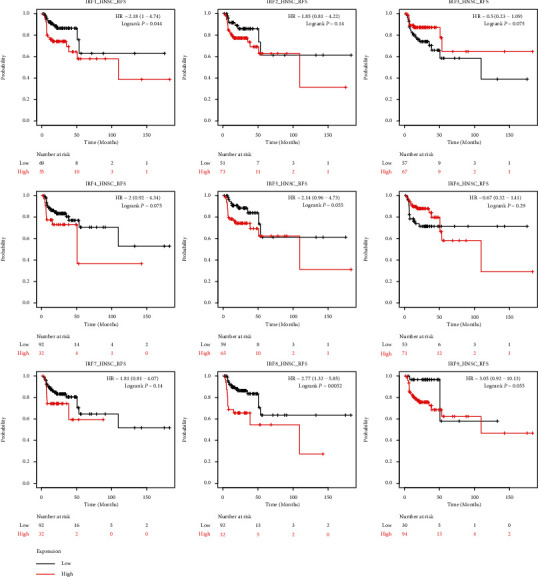
Kaplan–Meier survival curves for visualizing the association of relapse-free survival (RFS) and expression of IRF family genes in TCGA_HNSC data.

The TCGA database was applied for investigating the prognostic value of IRF family genes' mRNA expression in HNSC. Based on each IRF family gene's expression level, patients with TCGA-HNSC were divided into the IRF-high and IRF-low groups based on the median expression level.  Figure 3 shows that patients in the IRF1-high group (*p* = 0.045), IRF4-high group (*p* = 1.6*e* − 06), IRF5-high group (*p* = 0.039), IRF6-high group (*p* = 0.035), IRF8-high group (*p* = 0.0028), and IRF9-high group (*p* = 0.025) were associated with better survival in the TCGA-HNSC cohort (*p* = 0.025) (Figure 5). However, the other IRF family genes (IRF2 (*p* = 0.17), IRF3 (*p* = 0.11), and IRF7 (*p* = 0.32)) did not show prognostic values in the TCGA-HNSC cohort.  Figure 6 shows that the higher mRNA expression of IRF1 and IRF8 was correlated with poor relapse-free survival (*p* < 0.05), while the mRNA overexpression of the other IRF family genes (IRF2, IRF3, IRF4, IRF5, IRF6, and IRF7) did not significantly affect the overall survival in HNSC cases.

### 3.7. The Expression of IRF Family Genes in Different Tumor Stages of HNSC Patients

**Figure 7:**
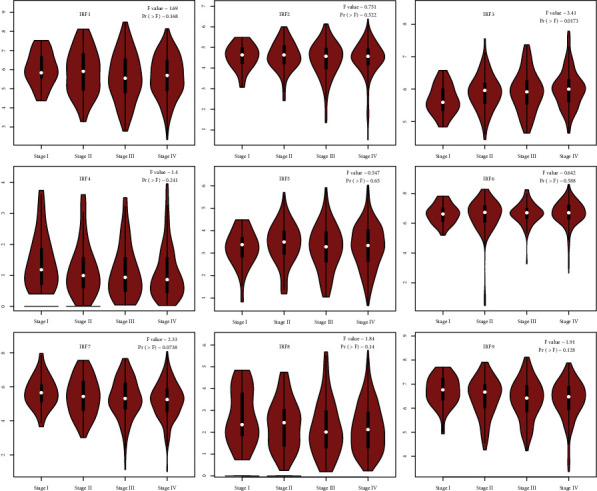
Pathological stage plot demonstrating the expression of a specific member of the IRF family at various tumor stages in HNSC patients.

The expression of members of the IRF family was examined in various stages of HNSC. IRF3 expression levels differed significantly between tumor stages (*p* = 0.0173 < 0.05), whereas the expression levels of the other IRFs did not alter significantly among tumor stages (Figure 7).

### 3.8. The PPI Network Construction

**Figure 8:**
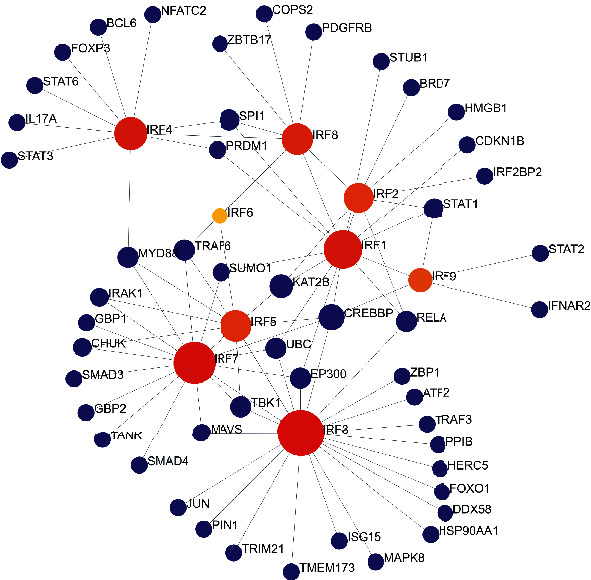
The generic protein-protein network obtained from the NetworkAnalyst webtool.

Figure 8 and Table S1 show that 22 genes interacted with IRF3, 17 genes interacted with IRF7, 14 genes interacted with IRF1, 10 genes interacted with IRF4, 9 genes interacted with IRF8, 9 genes interacted with IRF5, 8 genes interacted with IRF2, and 5 genes interacted with IRF9. IRF3 interacted with the greatest number of genes. The 50 genes interacting with IRFs were considered to be interacted genes and will be used for the subsequent analysis.

### 3.9. Identification of IRFs' Correlating Genes by GEPIA

By using the similar gene detection module of GEIPIA webtool, the top 200 genes with similar expression pattern with IRFs in HNSC were identified. Table S2 shows that the top 10 genes highly correlated with IRF family genes signature are as follows: IRF1, TIGIT, ICOS, IL2RB, SLA2, AKNA, FMNL1, CD2, FGD2, and ARHGAP30.

### 3.10. The Overlapping Genes between Interacted Genes and Correlated Genes

**Figure 9:**
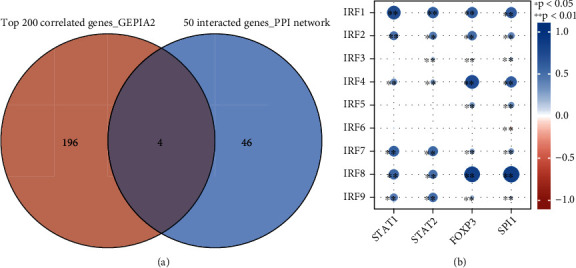
The overlapping between top 200 correlated genes and 50 interacted genes in the PPI network.

The Venn diagram in Figure 9(a) shows that 4 genes were at the intersection between 50 interacted genes in the PPI network and 200 correlated genes identified by GEPIA. These four genes are as follows: STAT1, STAT2, FOXP3, and SPI1. The correlation analysis results in Figure 9(b) and Table 2 showed that the majority of gene pairings had a substantial positive correlation. Between IRF6 and SPI1, a negative association was detected (*r* = −0.148, *p* < 0.001). IRF8 and SPI1 had the strongest positive connection (*r* = 0.864, *p* < 0.001).

### 3.11. Identification of IRFs Involved in Biological Functions

**Figure 10:**
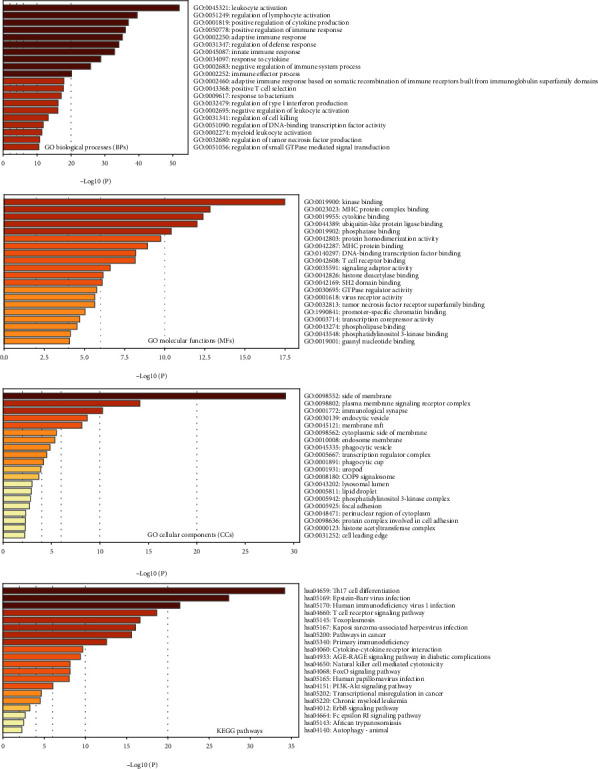
The results of functional enrichment analysis performed using the Metascape web portal.

The 50 IRFs-interacting genes and 200 IRFs-correlated genes were integrated into 246 genes.  Figure 10 shows that these 246 genes were implicated in immune cell-related biological processes (BPs) (e.g., leukocyte activation, regulation of lymphocyte activation, positive T cell selection, and negative regulation of leukocyte activation) and cytokine-related BPs (e.g., positive regulation of cytokine production, response to cytokine, and regulation of cytokine tumor necrosis factor (TNF) production). In addition, these 246 genes were significantly enriched in multiple molecular functions (MFs), for example, cytokine binding, TNF receptor superfamily binding, T cell receptor binding, and virus receptor activity. Furthermore, these 246 genes were implicated in several cellular components (CCs), for instance, focal adhesion, immunological synapse, and phosphoinositide 3-kinase (PI3K) complex. Most importantly, several KEGG pathways were found to be enriched, e.g., Th17 cell differentiation, T cell receptor signaling pathway, cytokine-cytokine receptor interaction, Natural killer (NK) cell-mediated cytotoxicity, FOXO signaling, PI3K-Akt signaling, and ErbB signaling.

### 3.12. The Correlation between IRF Expression and Immune Cells in HNSC

**Figure 11:**
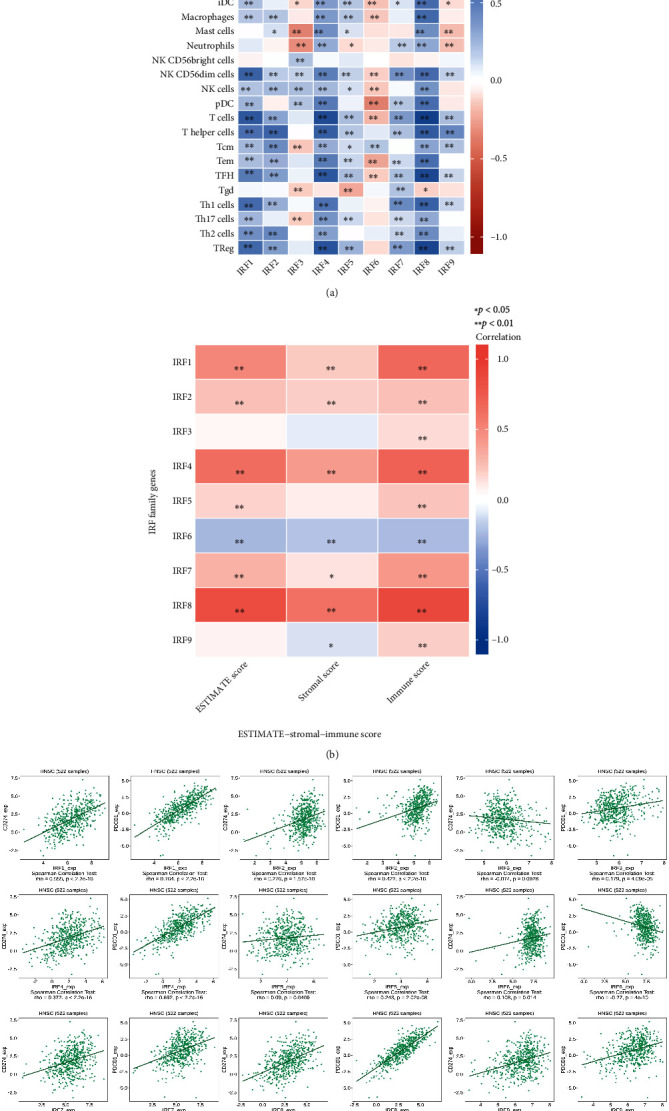
The tumor immunity analysis results regarding IRF family genes in HNSC. (a) The heatmap showing the relations between tumor-infiltrating immune cells and IRF family genes. (b) The correlation between IRF family genes and ESTIMATE-Stromal-Immune score in HNSC. (c) TISIB web portal for examining how immunoinhibitors (CD274 and PDCD1) were regulated by IRF family genes.

Figure 11(a) shows that most correlations between IRF gene members and TIICs were positive. The correlations between IRF6 and most TIICs (e.g., B cells, CD8 T cells, cytotoxic cells, dendritic cells, immature dendritic cells, macrophages, NK CD56dim cells, NK cells, plasmacytoid dendritic cells, T cells, T effector memory, T follicular helper, and T regulatory cells) were negative. The strongest positive connection (*r* = 0.849, *p* < 0.001) was seen between IRF8 and T cells. IRF6 and plasmacytoid dendritic cells had the strongest negative connection (*r* = −0.36, *p* < 0.001).

### 3.13. The Correlation between IRF Family Genes and ESTIMATE-Stromal-Immune Score

Figure 11(b) and Table 3 show that majority of correlations were found to be positive. The highest positive correlation was found to be between IRF8 and Immune score (*r* = 0.874, *p* = 1.09*E* − 158). The highest negative correlation was found to be between IRF6 and ESTIMATE score (*r* = −0.236, *p* = 8.75*E* − 08).

### 3.14. Relations between Each IRF Gene and Two Immunoinhibitor Genes

Figure 11(c) shows that the most correlations between IRFs and two immunoinhibitor genes (CD274 and PDCD1) were positive. The strongest positive connection (*r* = 0.764, *p* = 2.2*e* − 16) was reported between IRF1 and PDCD1. IRF6 and PDCD1 had the negative connection (*r* = −0.27, *p* = 4*e* − 10).

## 4. Discussion

The main findings of the current research showed that IRF family genes were involved in several signaling pathways, e.g., FOXO signaling pathway, PI3K-Akt signaling pathway, and ErbB signaling pathway. IRF family genes were significantly implicated in tumor immunity by being mainly positively correlated with tumor-infiltrating immune cells and regulating tumor immunosuppression.

Figure 9(b) shows that the majority of IRF family genes were positively correlated with their interacting and correlating four genes (STAT1, STAT2, FOXP3, and SPI1) in head and neck cancer. A significant role of the TAT1 and STAT2 proteins lies in an important role in interferon (IFN) signaling and cellular antiviral responses and adaptive immunity [9]. STAT1 and STAT2 proteins were found to associate with IRF9 to form a heterotrimeric transcription factor complex known as ISGF3 [15]. This should be the reason leading to the positive correlation between STAT1-2 and IRF9. The forkhead/winged-helix family transcriptional repressor FOXP3 is a surface marker specifically expressed in CD4+CD25+ Treg cells and plays a pivotal role in regulating its development and differentiation [16]. A previous study found that IRF1 was found to negatively regulate the function of CD4+CD25+ Treg cells by directly and specifically repressing the expression of FOXP3 gene, suggesting the negative correlation between IRF1 and FOXP3 in tumor immunity [17]. However, the prediction of our computational biology analysis showed a positive correlation between IRF1 and FOXP3 in HNSC. The transcription factor SPI1, as a protein of ETS family, is a surface marker expressed in macrophages and neutrophils [18]. The present research showed that SPI1 was positively correlated with the majority of IRF family genes except for IRF6. IRF4 and IRF8 were found to cooperate with SPI1 and thereby upregulate the expression of pro-inflammatory genes (e.g., CD20, Ig light chain enhancers, IL-18, and IL-1*β*) [19–21]. SPI1-IRF coactivating complexes such as IRF4-SP1 and IRF8-SPI1 were found to bind to a SPI1-IRF composite element in the promoter of the proinflammatory cytokine IL-1*β* [22]. Based on the role of interleukin-1*β* (IL-1*β*) in modulating the tumor microenvironment and further promoting tumor growth, the SPI1-IRF coactivating complexes might play a role in contributing to the formation of an inflammatory tumor microenvironment.

The functional enrichment analysis showed that IRF family genes' 246 correlated and interacted genes were significantly involved in several KEGG signaling pathways including FOXO signaling pathway, PI3K-Akt signaling pathway, and ErbB signaling pathway. A previous conducted by Yuan et al. identified IRF7 to be an important target gene of FOXO3 by carrying out genome-wide location analysis and gene deletion experiments [23]. FOXO3 as a member of the forkhead family was shown to negatively regulate a subset of antiviral genes (e.g., Gbp2, Ccl5, Ifit1, Irf7, and Oasl1) [24]. A regulatory circuit formed by FOXO3, IRF7, and IFN-I was found to limit inflammatory consequences resulting from antiviral responses [25]. In addition, the relationship of IRF family genes and PI3K/Akt pathway has been demonstrated by previous evidence [24]. Dhamanage et al. discovered that activation of the PI3K/Akt signaling pathway is required for IRF-7 translocation from the cytoplasm to the nucleus in plasmacytoid dendritic cells [26, 27]. The PI3K/Akt pathway was shown to diminish IRF-3-dependent promoter activity and impede dimerization of IRF-3, thereby further resulting in the loss of host antiviral activity [26]. The ErbB family of proteins consists of four receptor tyrosine kinases (e.g., Her1 (EGFR, ErbB1), Her2 (Neu, ErbB2), Her3 (ErbB3), and Her4 (ErbB4)) that share structural homology with the epidermal growth factor receptor (EGFR) [27]. The upregulation of IRF1 was found to be induced by the activation of EGFR signaling was manifested in an EGF dose-dependent manner. The induced expression of tumor suppressor IRF1 was found to alert antitumor immunity by activating immune effector cells and inhibiting cell proliferation of human cancer cells [28].

In terms of IRF family genes' relationship to tumor immunity, the present research revealed that the IRF family gene expression was associated with many types of immune cells, for example, NK cells, Treg cells, Th1 cells, macropahages, and Th17 cells. Our research showed that IRF3 was positively correlated with NK cells in HNSC (Figure 8(a)). In accordance with such findings, a previous study conducted by Andersen et al. found that exosomes derived from head and neck cancer cells were able to increase the expression of interferon regulatory factor 3 (IRF3) and further promoted the function of NK cells with respect to proliferation, cytotoxicity, and the release of perforin and granzyme M [29, 30]. Our research showed that IRF1 was significantly positively correlated with Treg cells and Th1 cells. In a related finding, a previous study conducted by Chen et al. showed that IRF1 exhibited immunomodulatory activities by controlling Treg depletion and governing Th1 polarization [31]. Additionally, it has been well established that a high infiltration of macrophages is associated with decreased overall survival and metastatic progression. Our research found that IRF8 was positively correlated with macrophages in HSNC. A study by Buccione et al. showed that IRF8 was able to transcriptionally modulate the response of macrophages by driving macropshages towards a more protumor and prometastasis phenotype [32]. Additionally, the present study found that IRF8 was positively correlated with Th17 in HNSC. However, a previous study using experimental colitis showed a negative correlation between IRF8 and Th17. This study found that IRF8 is required for Th17 cell differentiation to be suppressed: IRF8 transduction decreased the expression of genes linked with Th17; and vice versa, the lack of IRF8 was able to enhance the immune response induced by Th17 [33]. The tumor microenvironment of HNSC was found to strongly induce Th17 cells through cytokines; and in another way, the presence of Th17 cells was able to promote the proliferation and angiogenesis of HNSCC [34]. Based on the promoting role of Th17 in the carcinogenesis of HNSCC as well as the promoting role of IRF8 in HNSCC, the correlation between Th17 and IRF8 should be speculated to be positive; however, it still needs to be verified in the future experimental investigations.

Regarding the involvement of IRF family genes in tumor immunosuppression, the present study found that IRF family genes were significantly correlated with the immune checkpoint genes (PDL1 and its receptor PDCD1) in HNSC. The correlation between PDL1 and interferon-*γ* (IFN*γ*) has been demonstrated by showing that IFN upregulated PDL1 in a variety of HNSC cell lines regardless of HPV infection. The high link between IFN and PDL1 is explained by the fact that an antitumor cellular immune response mediated by natural killer (NK) cells and CD8+ tumor-infiltrating lymphocytes (TILs) produces IFN, which in turn induces PDL1 expression on tumor cells [35]. However, the current research regarding the correlation between PDL1 and IRF family genes especially in HNSC is lacking. According to the authors' knowledge, there is a paucity of evidence showing the PDL1 and IRF family genes in other types of cancers. For instance, a previous study investigating lung cancer found that interferon-*β* (IFN-*β*) induced the upregulation of PDL1 in lung cancer cells via the activation of the IRF9 pathway [36]. Although a different tumor type, the current research also found a positive correlation between PDL1 and IRF9 in HNSC; however, such finding needs to be verified by future experimental research. Another research investigating hepatocellular carcinoma (HCC) also found that IRF-1 was able to upregulate the IFN-*γ*-induced PDL1 mRNA and protein expressions, indicating a positive correlation between IRF1 and PDL1 [37]. In accordance with these findings, the present study also showed that PDL1 was positively correlated with IRF1 in HNSC. Additionally, the current study showed that IRF3 was not associated with PDL1 in HNSC (*p* = 0.0928); however, a previous research found that IRF3 promoted the induction of PDL1 by forming a transcriptional complex with NF-*κ*B/p65, and further resulting in the solar ultraviolet radiation- (UVR-) induced immune suppression [38].

In summary, the current research provided a comprehensive view regarding the implication of IRF family genes in HNSC from a variety of aspects, for example, expression pattern, prognostic values, relationship with clinical stages, interacting and correlating genes, enriched biological functions, involved signaling pathways, correlation with tumor cells, and classic immune checkpoint genes. The current research has identified directions towards a better understanding of mechanisms of IRF family genes in HNSC, and these findings based on computational prediction warrant experimental work for validation.

## 5. Conclusion

The upregulation of the IRF1, IRF4, IRF5, IRF6, IRF8, and IRF9 genes was identified as a potential prognostic indicator in HNSC. IRF family genes played a tumor-promoting role in HNSC by involving several pathways including Th17 cell differentiation, T cell receptor signaling pathway, cytokine-cytokine receptor interaction, NK cell-mediated cytotoxicity, FOXO signaling, PI3K-Akt signaling, and ErbB signaling. Given such findings, the genes of the IRF family should be considered possible therapeutic targets in the treatment of head and neck cancer.

## Figures and Tables

**Table 1:** The correlation among IRF family genes in HNSC, analyzed by Pearson correlation coefficient analysis.

IRF family genes	IRF1		IRF2		IRF3		IRF4		IRF5		IRF6		IRF7		IRF8		IRF9	
	*r* value	*p* value	*r* value	*p* value	*r* value	*p* value	*r* value	*p* value	*r* value	*p* value	*r* value	*p* value	*r* value	*p* value	*r* value	*p* value	*r* value	*p* value
IRF1	1.000	<0.001	0.493	<0.001	0.274	<0.001	0.451	<0.001	0.223	<0.001	0.089	0.046	0.471	<0.001	0.632	<0.001	0.414	<0.001
IRF2	0.493	<0.001	1.000	<0.001	0.005	0.916	0.389	<0.001	0.371	<0.001	0.316	<0.001	0.057	0.204	0.436	<0.001	0.324	<0.001
IRF3	0.274	<0.001	0.005	0.916	1.000	<0.001	-0.008	0.859	-0.022	0.625	-0.045	0.314	0.273	<0.001	0.103	0.021	0.253	<0.001
IRF4	0.451	<0.001	0.389	<0.001	-0.008	0.859	1.000	<0.001	0.209	<0.001	-0.074	0.098	0.153	<0.001	0.767	<0.001	0.220	<0.001
IRF5	0.223	<0.001	0.371	<0.001	-0.022	0.625	0.209	<0.001	1.000	<0.001	0.205	<0.001	-0.107	0.017	0.307	<0.001	0.100	0.025
IRF6	0.089	0.046	0.316	<0.001	-0.045	0.314	-0.074	0.098	0.205	<0.001	1.000	<0.001	-0.060	0.176	-0.124	0.005	0.124	0.005
IRF7	0.471	<0.001	0.057	0.204	0.273	<0.001	0.153	<0.001	-0.107	0.017	-0.060	0.176	1.000	<0.001	0.305	<0.001	0.340	<0.001
IRF8	0.632	<0.001	0.436	<0.001	0.103	0.021	0.767	<0.001	0.307	<0.001	-0.124	0.005	0.305	<0.001	1.000	<0.001	0.250	<0.001
IRF9	0.414	<0.001	0.324	<0.001	0.253	<0.001	0.220	<0.001	0.100	0.025	0.124	0.005	0.340	<0.001	0.250	<0.001	1.000	<0.001

**Table 2:** The correlation between IRFs and four interacted and correlated genes, based on the Pearson correlation coefficient analysis.

	STAT1		STAT2		FOXP3		SPI1	
	*r* value	*p* value	*r* value	*p* value	*r* value	*p* value	*r* value	*p* value
IRF1	0.729	<0.001	0.61	<0.001	0.607	<0.001	0.601	<0.001
IRF2	0.483	<0.001	0.436	<0.001	0.51	<0.001	0.382	<0.001
IRF3	0.087	0.052	0.217	<0.001	0.116	0.009	0.2	<0.001
IRF4	0.367	<0.001	0.283	<0.001	0.738	<0.001	0.604	<0.001
IRF5	0.045	0.319	0.043	0.332	0.312	<0.001	0.343	<0.001
IRF6	0.107	0.016	0.064	0.152	-0.009	0.841	-0.148	<0.001
IRF7	0.575	<0.001	0.543	<0.001	0.289	<0.001	0.321	<0.001
IRF8	0.556	<0.001	0.49	<0.001	0.836	<0.001	0.864	<0.001
IRF9	0.438	<0.001	0.508	<0.001	0.234	<0.001	0.178	<0.001

**Table 3:** The correlation between IRF family genes and ESTIMATE-Stromal-Immune score in HNSC.

IRF family genes	ESTIMATE score		Stromal score		Immune score	
	*r* value	*p* value	*r* value	*p* value	*r* value	*p* value
IRF1	0.5	4.38*E* − 33	0.198	7.98*E* − 06	0.667	5.93*E* − 66
IRF2	0.239	5.82*E* − 08	0.182	4.21*E* − 05	0.239	5.67*E* − 08
IRF3	0.042	0.353	-0.068	0.13	0.133	0.003
IRF4	0.633	1.49*E* − 57	0.403	5.19*E* − 21	0.705	1.14*E* − 76
IRF5	0.165	2.08*E* − 04	0.073	0.103	0.213	1.46*E* − 06
IRF6	-0.236	8.75*E* − 08	-0.193	1.35*E* − 05	-0.224	4.05*E* − 07
IRF7	0.303	3.86*E* − 12	0.101	0.023	0.422	4.25*E* − 23
IRF8	0.844	2.46*E* − 137	0.609	2.30*E* − 52	0.874	1.09*E* − 158
IRF9	0.054	0.225	-0.101	0.023	0.185	2.98*E* − 05

## Data Availability

The data that support the findings of this study are available from the corresponding author upon reasonable request.

## Supplementary Materials

Supplementary Materials Table S1: the degree and betweeness values of 59 nodes in the PPI network, ranking by the descending order of the degree value. Table S2: the PCC values of the top 200 IRF-correlated genes identified by using the GEPIA webtool.
