# Supplementary material for: Interferon Regulatory Factor Family Genes: At the Crossroads between Immunity and Head and Neck Squamous Carcinoma
Source: Dis Markers. 2022 May 26;2022:2561673. doi: 10.1155/2022/2561673 (PMC9162818; doi:10.1155/2022/2561673)
Supplement: Supplementary Materials — Table S1: the degree and betweeness values of 59 nodes in the PPI network, ranking by the descending order of the degree value. Table S2: the PCC values of the top 200 IRF-correlated genes identified by using the GEPIA webtool. [file 2561673.f1.docx]

**Supplementary Materials**

**Table S1.** The degree and between values of 59 nodes in PPI network, ranking by the descending order of the degree value.

| **Id** | **Label** | **Degree** | **Betweenness** | **Expression** |
| --- | --- | --- | --- | --- |
| 3661 | IRF3 | 22 | 742.58 | 0 |
| 3665 | IRF7 | 17 | 492.34 | 0 |
| 3659 | IRF1 | 14 | 380.96 | 0 |
| 3662 | IRF4 | 10 | 342.64 | 0 |
| 3394 | IRF8 | 9 | 272.62 | 0 |
| 3663 | IRF5 | 9 | 187.95 | 0 |
| 3660 | IRF2 | 8 | 193.75 | 0 |
| 1387 | CREBBP | 5 | 134.1 | 0 |
| 10379 | IRF9 | 5 | 117.93 | 0 |
| 8850 | KAT2B | 4 | 60.48 | 0 |
| 4615 | MYD88 | 3 | 197.54 | 0 |
| 5970 | RELA | 3 | 103.29 | 0 |
| 7189 | TRAF6 | 3 | 56.79 | 0 |
| 2033 | EP300 | 3 | 39 | 0 |
| 7316 | UBC | 3 | 39 | 0 |
| 6688 | SPI1 | 3 | 25.68 | 0 |
| 6772 | STAT1 | 3 | 6 | 0 |
| 29110 | TBK1 | 3 | 1.52 | 0 |
| 639 | PRDM1 | 2 | 25.68 | 0 |
| 3664 | IRF6 | 2 | 13.93 | 0 |
| 7341 | SUMO1 | 2 | 9.18 | 0 |
| 3654 | IRAK1 | 2 | 1.52 | 0 |
| 1147 | CHUK | 2 | 1.52 | 0 |
| 57506 | MAVS | 2 | 0 | 0 |
| 10273 | STUB1 | 1 | 0 | 0 |
| 1027 | CDKN1B | 1 | 0 | 0 |
| 5159 | PDGFRB | 1 | 0 | 0 |
| 1386 | ATF2 | 1 | 0 | 0 |
| 5300 | PIN1 | 1 | 0 | 0 |
| 6737 | TRIM21 | 1 | 0 | 0 |
| 51191 | HERC5 | 1 | 0 | 0 |
| 5479 | PPIB | 1 | 0 | 0 |
| 10010 | TANK | 1 | 0 | 0 |
| 604 | BCL6 | 1 | 0 | 0 |
| 3605 | IL17A | 1 | 0 | 0 |
| 6774 | STAT3 | 1 | 0 | 0 |
| 6778 | STAT6 | 1 | 0 | 0 |
| 50943 | FOXP3 | 1 | 0 | 0 |
| 359948 | IRF2BP2 | 1 | 0 | 0 |
| 3146 | HMGB1 | 1 | 0 | 0 |
| 3455 | IFNAR2 | 1 | 0 | 0 |
| 6773 | STAT2 | 1 | 0 | 0 |
| 9318 | COPS2 | 1 | 0 | 0 |
| 340061 | TMEM173 | 1 | 0 | 0 |
| 4088 | SMAD3 | 1 | 0 | 0 |
| 3320 | HSP90AA1 | 1 | 0 | 0 |
| 4089 | SMAD4 | 1 | 0 | 0 |
| 5599 | MAPK8 | 1 | 0 | 0 |
| 2634 | GBP2 | 1 | 0 | 0 |
| 2633 | GBP1 | 1 | 0 | 0 |
| 81030 | ZBP1 | 1 | 0 | 0 |
| 3725 | JUN | 1 | 0 | 0 |
| 7709 | ZBTB17 | 1 | 0 | 0 |
| 9636 | ISG15 | 1 | 0 | 0 |
| 2308 | FOXO1 | 1 | 0 | 0 |
| 23586 | DDX58 | 1 | 0 | 0 |
| 7187 | TRAF3 | 1 | 0 | 0 |
| 29117 | BRD7 | 1 | 0 | 0 |
| 4773 | NFATC2 | 1 | 0 | 0 |

**Table S2.** The PCC values of the top 200 IRFs-correlated genes identified by using GEPIA webtool.

| **Gene Symbol** | **Gene ID** | **PCC** |
| --- | --- | --- |
| IRF1 | ENSG00000125347.13 | 0.8 |
| TIGIT | ENSG00000181847.11 | 0.79 |
| ICOS | ENSG00000163600.12 | 0.78 |
| IL2RB | ENSG00000100385.13 | 0.77 |
| SLA2 | ENSG00000101082.13 | 0.76 |
| AKNA | ENSG00000106948.16 | 0.76 |
| FMNL1 | ENSG00000184922.13 | 0.76 |
| CD2 | ENSG00000116824.4 | 0.75 |
| FGD2 | ENSG00000146192.14 | 0.75 |
| ARHGAP30 | ENSG00000186517.13 | 0.75 |
| CCR5 | ENSG00000160791.13 | 0.75 |
| GIMAP2 | ENSG00000106560.10 | 0.75 |
| CD3E | ENSG00000198851.9 | 0.74 |
| TNFRSF1B | ENSG00000028137.16 | 0.74 |
| CXCR6 | ENSG00000172215.5 | 0.74 |
| TRAC | ENSG00000277734.4 | 0.74 |
| SNX20 | ENSG00000167208.14 | 0.73 |
| RP11-327F22.2 | ENSG00000261644.1 | 0.73 |
| ITGAL | ENSG00000005844.17 | 0.73 |
| TRAFD1 | ENSG00000135148.11 | 0.73 |
| FOXP3 | ENSG00000049768.14 | 0.73 |
| SIRPG | ENSG00000089012.14 | 0.73 |
| SASH3 | ENSG00000122122.9 | 0.73 |
| MYO1F | ENSG00000142347.16 | 0.73 |
| PARP14 | ENSG00000173193.13 | 0.72 |
| NLRC3 | ENSG00000167984.16 | 0.72 |
| WAS | ENSG00000015285.10 | 0.72 |
| IL10RA | ENSG00000110324.9 | 0.72 |
| DOCK2 | ENSG00000134516.15 | 0.72 |
| ITK | ENSG00000113263.12 | 0.72 |
| PDCD1 | ENSG00000188389.10 | 0.72 |
| UBASH3A | ENSG00000160185.13 | 0.72 |
| SLA | ENSG00000155926.13 | 0.72 |
| IL12RB1 | ENSG00000096996.15 | 0.72 |
| ARHGAP25 | ENSG00000163219.11 | 0.72 |
| ABI3 | ENSG00000108798.8 | 0.72 |
| SPN | ENSG00000197471.11 | 0.72 |
| CLEC2D | ENSG00000069493.14 | 0.72 |
| RP11-1094M14.8 | ENSG00000267369.1 | 0.72 |
| RNF213 | ENSG00000173821.19 | 0.72 |
| AKAP5 | ENSG00000179841.8 | 0.71 |
| SLFN12L | ENSG00000205045.8 | 0.71 |
| NCKAP1L | ENSG00000123338.12 | 0.71 |
| UBA7 | ENSG00000182179.10 | 0.71 |
| SELPLG | ENSG00000110876.9 | 0.71 |
| GPR65 | ENSG00000140030.5 | 0.71 |
| APOL3 | ENSG00000128284.19 | 0.71 |
| CXCR3 | ENSG00000186810.7 | 0.71 |
| PTPRC | ENSG00000081237.18 | 0.71 |
| RP11-1094M14.5 | ENSG00000267074.1 | 0.71 |
| APOBEC3G | ENSG00000239713.7 | 0.71 |
| LAP3 | ENSG00000002549.12 | 0.7 |
| CD96 | ENSG00000153283.12 | 0.7 |
| TBX21 | ENSG00000073861.2 | 0.7 |
| CIITA | ENSG00000179583.17 | 0.7 |
| FLT3LG | ENSG00000090554.12 | 0.7 |
| PYHIN1 | ENSG00000163564.14 | 0.7 |
| SAMHD1 | ENSG00000101347.8 | 0.7 |
| CD53 | ENSG00000143119.12 | 0.7 |
| PREX1 | ENSG00000124126.13 | 0.7 |
| CYTH4 | ENSG00000100055.20 | 0.7 |
| BTN3A1 | ENSG00000026950.16 | 0.7 |
| CD74 | ENSG00000019582.14 | 0.69 |
| APBB1IP | ENSG00000077420.15 | 0.69 |
| SAMSN1 | ENSG00000155307.17 | 0.69 |
| CD4 | ENSG00000010610.9 | 0.69 |
| APOL6 | ENSG00000221963.5 | 0.69 |
| GIMAP4 | ENSG00000133574.9 | 0.69 |
| TRANK1 | ENSG00000168016.13 | 0.69 |
| GPRIN3 | ENSG00000185477.4 | 0.69 |
| STAT1 | ENSG00000115415.18 | 0.69 |
| IKZF1 | ENSG00000185811.16 | 0.69 |
| AD000671.6 | ENSG00000267120.3 | 0.69 |
| DOK2 | ENSG00000147443.12 | 0.69 |
| HAPLN3 | ENSG00000140511.11 | 0.69 |
| IFI30 | ENSG00000216490.3 | 0.69 |
| HAVCR2 | ENSG00000135077.8 | 0.69 |
| IL18BP | ENSG00000137496.17 | 0.69 |
| CD6 | ENSG00000013725.14 | 0.69 |
| LILRB1 | ENSG00000104972.14 | 0.69 |
| ITGB7 | ENSG00000139626.15 | 0.69 |
| CXorf21 | ENSG00000120280.5 | 0.69 |
| ETV7 | ENSG00000010030.13 | 0.69 |
| LCP2 | ENSG00000043462.11 | 0.69 |
| GNGT2 | ENSG00000167083.6 | 0.69 |
| TNFSF13B | ENSG00000102524.11 | 0.69 |
| HLA-DPA1 | ENSG00000231389.7 | 0.69 |
| SEPT6 | ENSG00000125354.22 | 0.69 |
| NUB1 | ENSG00000013374.15 | 0.69 |
| BIN2 | ENSG00000110934.10 | 0.68 |
| HNRNPA1P21 | ENSG00000228168.1 | 0.68 |
| CD3G | ENSG00000160654.9 | 0.68 |
| LINC00996 | ENSG00000242258.1 | 0.68 |
| CDC42SE2 | ENSG00000158985.13 | 0.68 |
| SH2D1A | ENSG00000183918.14 | 0.68 |
| JAKMIP1 | ENSG00000152969.16 | 0.68 |
| HLA-DRA | ENSG00000204287.13 | 0.68 |
| DOK3 | ENSG00000146094.13 | 0.68 |
| DOCK8 | ENSG00000107099.15 | 0.68 |
| RASAL3 | ENSG00000105122.12 | 0.68 |
| CD8A | ENSG00000153563.15 | 0.68 |
| CLNK | ENSG00000109684.14 | 0.68 |
| LAT2 | ENSG00000086730.16 | 0.68 |
| GRAP2 | ENSG00000100351.16 | 0.68 |
| LINC00426 | ENSG00000238121.5 | 0.68 |
| GMIP | ENSG00000089639.10 | 0.67 |
| CD7 | ENSG00000173762.7 | 0.67 |
| HLA-DMB | ENSG00000242574.8 | 0.67 |
| PIK3R5 | ENSG00000141506.13 | 0.67 |
| SAMD3 | ENSG00000164483.16 | 0.67 |
| GPSM3 | ENSG00000213654.9 | 0.67 |
| FTH1P22 | ENSG00000225079.2 | 0.67 |
| IL15RA | ENSG00000134470.19 | 0.67 |
| CYTIP | ENSG00000115165.9 | 0.67 |
| IRF2 | ENSG00000168310.10 | 0.67 |
| EVI2B | ENSG00000185862.6 | 0.67 |
| TRIM22 | ENSG00000132274.15 | 0.67 |
| TRBC2 | ENSG00000211772.8 | 0.67 |
| CD226 | ENSG00000150637.8 | 0.67 |
| TESPA1 | ENSG00000135426.14 | 0.67 |
| SSTR3 | ENSG00000278195.1 | 0.67 |
| TRBV25-1 | ENSG00000211751.7 | 0.67 |
| CXCR2P1 | ENSG00000229754.1 | 0.67 |
| ZC3H12D | ENSG00000178199.13 | 0.67 |
| HLA-DPB1 | ENSG00000223865.10 | 0.67 |
| MCOLN2 | ENSG00000153898.12 | 0.66 |
| ARHGDIB | ENSG00000111348.8 | 0.66 |
| SLAMF6 | ENSG00000162739.13 | 0.66 |
| SAMD9L | ENSG00000177409.11 | 0.66 |
| THEMIS2 | ENSG00000130775.15 | 0.66 |
| STAT5A | ENSG00000126561.16 | 0.66 |
| SLAMF1 | ENSG00000117090.14 | 0.66 |
| ARHGAP9 | ENSG00000123329.17 | 0.66 |
| FAM78A | ENSG00000126882.12 | 0.65 |
| RP11-75L1.2 | ENSG00000213443.2 | 0.65 |
| APOBEC3D | ENSG00000243811.7 | 0.65 |
| ZNF831 | ENSG00000124203.5 | 0.65 |
| IKZF3 | ENSG00000161405.16 | 0.65 |
| GVINP1 | ENSG00000254838.5 | 0.65 |
| TRBV20-1 | ENSG00000211747.3 | 0.65 |
| TFEC | ENSG00000105967.15 | 0.65 |
| TAP1 | ENSG00000168394.10 | 0.65 |
| TRAV8-6 | ENSG00000211795.3 | 0.65 |
| PRF1 | ENSG00000180644.6 | 0.65 |
| FASLG | ENSG00000117560.7 | 0.65 |
| GIMAP1-GIMAP5 | ENSG00000281887.2 | 0.65 |
| TNFAIP8L2 | ENSG00000163154.5 | 0.65 |
| ARHGAP15 | ENSG00000075884.12 | 0.65 |
| LAT | ENSG00000213658.10 | 0.65 |
| PPP1R16B | ENSG00000101445.9 | 0.65 |
| IL21R | ENSG00000103522.15 | 0.65 |
| CST7 | ENSG00000077984.5 | 0.65 |
| TMEM140 | ENSG00000146859.6 | 0.65 |
| DTX3L | ENSG00000163840.9 | 0.65 |
| CD3D | ENSG00000167286.9 | 0.65 |
| STAT2 | ENSG00000170581.13 | 0.65 |
| C9orf91 | ENSG00000157693.14 | 0.65 |
| TRGC1 | ENSG00000211689.6 | 0.65 |
| TNFRSF14 | ENSG00000157873.17 | 0.65 |
| P2RY10 | ENSG00000078589.12 | 0.65 |
| IGFLR1 | ENSG00000126246.9 | 0.65 |
| SPI1 | ENSG00000066336.11 | 0.64 |
| ACSL5 | ENSG00000197142.10 | 0.64 |
| TRAV29DV5 | ENSG00000211810.3 | 0.64 |
| NAIP | ENSG00000249437.7 | 0.64 |
| TRAV9-2 | ENSG00000211793.2 | 0.64 |
| LCK | ENSG00000182866.16 | 0.64 |
| AC116366.6 | ENSG00000234290.2 | 0.64 |
| CORO1A | ENSG00000102879.15 | 0.64 |
| SIGLEC10 | ENSG00000142512.14 | 0.64 |
| GIMAP7 | ENSG00000179144.4 | 0.64 |
| TIFAB | ENSG00000255833.1 | 0.64 |
| WDFY4 | ENSG00000128815.17 | 0.64 |
| BTK | ENSG00000010671.15 | 0.64 |
| CD48 | ENSG00000117091.9 | 0.64 |
| GPR174 | ENSG00000147138.1 | 0.64 |
| SCIMP | ENSG00000161929.14 | 0.64 |
| GAB3 | ENSG00000160219.11 | 0.64 |
| TAPBP | ENSG00000231925.11 | 0.64 |
| C5orf56 | ENSG00000197536.10 | 0.64 |
| GPR82 | ENSG00000171657.5 | 0.63 |
| ERAP1 | ENSG00000164307.12 | 0.63 |
| TRBV5-1 | ENSG00000211734.3 | 0.63 |
| TNFRSF9 | ENSG00000049249.8 | 0.63 |
| SFMBT2 | ENSG00000198879.11 | 0.63 |
| PRKCB | ENSG00000166501.12 | 0.63 |
| CCR8 | ENSG00000179934.6 | 0.63 |
| GIMAP1 | ENSG00000213203.2 | 0.63 |
| SIT1 | ENSG00000137078.8 | 0.63 |
| AC083949.1 | ENSG00000224875.2 | 0.63 |
| P2RY13 | ENSG00000181631.6 | 0.63 |
| CD80 | ENSG00000121594.11 | 0.63 |
| RNASE6 | ENSG00000169413.2 | 0.63 |
| ADAR | ENSG00000160710.15 | 0.63 |
| TOMM20P2 | ENSG00000255987.1 | 0.63 |
| MARCH1 | ENSG00000145416.13 | 0.63 |
| CMPK2 | ENSG00000134326.11 | 0.63 |
| B2M | ENSG00000166710.17 | 0.63 |
| SLC7A7 | ENSG00000155465.18 | 0.63 |
| PIK3R6 | ENSG00000276231.4 | 0.63 |
